# Long-Term Risk of Subsequent Malignant Neoplasms Among Childhood and Adolescent Lymphoma Survivors (1975-2013): A Population-Based Predictive Nomogram

**DOI:** 10.1093/oncolo/oyad112

**Published:** 2023-05-13

**Authors:** Junqi Liu, Qingzhu Zheng, Narasimha M Beeraka, Xiao Zhang, Tingxuan Li, Ruixia Song, Di Zhao, Ruitai Fan

**Affiliations:** Department of Radiation Oncology, The First Affiliated Hospital of Zhengzhou University, Erqi, Zhengzhou, People’s Republic of China; Department of Radiation Oncology, The First Affiliated Hospital of Zhengzhou University, Erqi, Zhengzhou, People’s Republic of China; Raghavendra Institute of Pharmaceutical Education and Research (RIPER), Anantapuramu, Chiyyedu, Andhra Pradesh, India; Department of Human Anatomy, I.M. Sechenov First Moscow State Medical University (Sechenov University), Moscow, Russian Federation; Department of Radiation Oncology, The First Affiliated Hospital of Zhengzhou University, Erqi, Zhengzhou, People’s Republic of China; Department of Radiation Oncology, The First Affiliated Hospital of Zhengzhou University, Erqi, Zhengzhou, People’s Republic of China; Department of Radiation Oncology, The First Affiliated Hospital of Zhengzhou University, Erqi, Zhengzhou, People’s Republic of China; Endocrinology Department, The First Affiliated Hospital of Zhengzhou University, Zhengzhou, People’s Republic of China; Department of Radiation Oncology, The First Affiliated Hospital of Zhengzhou University, Erqi, Zhengzhou, People’s Republic of China

**Keywords:** nomogram, secondary malignancies, childhood lymphoma, adolescent lymphoma

## Abstract

**Background:**

Studies are needed to assess risk factors pertinent to the incidence of secondary malignancies among childhood and adolescent lymphoma survivors. We aimed to identify risk factors pertinent to the incidence of secondary malignancies and subsequently establish a clinically practical predictive nomogram.

**Methods:**

A total of 5561 patients who were diagnosed with primary lymphoma below the age of 20 years between 1975 and 2013 and survived for at least 5 years were identified. Standardized incidence ratio (SIR) and excess risk (ER) analysis were performed by sex, age, and year when primary lymphoma was diagnosed, sites and types of primary lymphoma, and therapy strategies. Univariable and multivariable logistic regression were used to identify independent risk factors for adolescent and childhood lymphoma-related secondary malignancies. Based on 5 factors (age, time from lymphoma diagnosis, gender, lymphoma type, and therapy), a nomogram for predicting the risk of a secondary malignancy for patients with childhood and adolescent primary lymphoma was established.

**Results:**

Among 5561 lymphoma survivors, 424 developed a secondary malignancy. Females (SIR = 5.34, 95% CI, 4.73-5.99; ER = 50.58) exhibited a higher SIR and ER than males (SIR = 3.28, 95% CI, 2.76-3.87; ER = 15.53). Blacks were at a higher risk than Caucasians or others. Nodular lymphocyte-predominant Hodgkin lymphoma survivors exhibited typically high SIR (13.13, 95% CI, 6-24.92) and ER (54.79) among all lymphoma classifications. Lymphoma survivors who underwent radiotherapy, whether they received chemotherapy or not, had typically higher SIR and ER. Among all types of secondary malignancies, “bone and joint neoplasms” (SIR = 11.07, 95% CI, 5.52-19.81) and “soft tissue neoplasms” (SIR = 12.27, 95% CI, 7.59-18.76) presented significantly high SIR whereas “breast cancer” and “endocrine cancer” associated with higher ER. The median diagnosis age of secondary malignancies was 36 years old, and the median time interval between the diagnosis of two malignancies was 23 years. A nomogram was constructed to predict the risk of secondary malignancies in patients diagnosed with primary lymphoma before 20 years of age. After internal validation, the AUC and C-index of the nomogram are 0.804 and 0.804, respectively.

**Conclusion and Relevance:**

The established nomogram provides a convenient and reliable tool for predicting the risk of a secondary malignancy among childhood and adolescent lymphoma survivors, concluding significant concern for lymphoma survivors with high-risk estimates.

## Teaser

This article assesses risk factors pertinent to the incidence of secondary malignancies among childhood and adolescent lymphoma survivors and reports a clinically practical predictive nomogram.

**Implications for Practice**

The authors developed a useful nomogram to specifically predict secondary malignancy incidence risk in primary lymphoma patients. This nomogram has great predictive power for lymphoma-related secondary malignancy risk based on the receiver operating characteristic curves and the calibration curves. This study identified several variables to develop a nomogram for predicting the incidence risk of secondary malignancy in patients with lymphoma.

## Introduction

Life expectancy is significantly shorter among individuals who survived childhood cancer.^1^ Recent advancements in therapeutic modalities have optimally increased the overall 5-year survival rate to 80% among individuals suffering from pediatric cancers.^2^ Hodgkin lymphoma is the most common cancer diagnosed in children and adolescents.^3^ It has been reported that lymphoma survivors may experience a 1.6 to 4.3 times higher incidence of second malignancies compared to pediatric patients who had solid tumors and were cured.^4^ A study in the Netherlands reported the incidence risk of secondary cancers in Hodgkin lymphoma patients diagnosed at the age of 15-50 years and survived for at least 5 years to be 13.9% at 30 years.^5^ Moreover, previous studies have reported the significant influence of radiotherapy or chemotherapy in the development of second malignancies among adolescents who were treated for Hodgkin lymphoma or non-Hodgkin lymphoma (NHL).^6,7^ Nowadays, the incidence of secondary malignancies remains a major problem in long-term lymphoma survivors, hence risk factors related to incidence should be identified to develop a suitable screening strategy.

Furthermore, studies demonstrated that adolescent cancer survivors had a higher absolute risk of secondary malignancies compared to younger or older cancer survivors.^5^ Nomograms could be used to explore prognostic strategies by ascertaining the patient’s overall survival information.^8^ There is no reported predictive nomogram evaluating the risk of secondary malignancies for childhood and adolescent lymphoma survivors. The current study analyzed the risk of developing secondary malignancies among childhood and adolescent lymphoma survivors depending on factors such as gender, age at diagnosis, year of diagnosis, incidence site, histological type, and treatment exposure data. Such a nomogram could benefit clinicians and oncologists to guide the ongoing surveillance of high-risk survivors.

## Methods

### Study Population

Patient data were downloaded from a database of the Surveillance, Epidemiology, and End Results (SEER) project, the SEER 9 1975-2018 (November 2020 sub), race (White/Black/other [W/B/O]), containing 9 SEER registries and covering approximately 9% of the USA population.^9^ Ethical approval was waived for this study as the data are publicly available.

The selection criteria for this study:

(1) patients diagnosed with primary lymphoma less than 20 years old between January 1, 1975 and December 31, 2013
(2) lymphoma diagnosis was pathologically confirmed
(3) survival time of more than 5 years

The exclusion criteria: (1) the Ann Arbor Stage of lymphoma is unknown, (2) secondary malignancy is lymphoma, and (3) diagnosed with a secondary malignancy within 1 year after a primary lymphoma diagnosis. In this study, secondary malignancy is defined as a different type of malignancy that develops in the same individual 1 year after the initial cancer diagnosis; this strategy prevents recognizing the relapse of “primary lymphoma” as “subsequent malignancy” and helps to minimize the chances of surveillance bias, as the likelihood of incidental cancer detection could be higher immediately following the initial cancer diagnosis.^10^ The primary lymphoma and secondary malignancy were classified according to ICD-O-3.^11,12^ Follow-up ended at the subsequent diagnosis of malignancies or death after December 2018 whichever occurred primarily.^13^

### Relative and Absolute Risk Analysis

According to the data obtained from the 9 registries of the SEER database, the standardized incidence ratio (SIR) is based on the “observed count,” which refers to the number of lymphoma survivors who developed secondary malignancies for a selected cohort, and the “expected count,” which refers to the hypothetical number of secondary malignancies calculated based on the “accumulation of person-years at risk” for the selected lymphoma survivors and the “incidence of a specific malignancy in the USA general population” adjusted by gender, race, type of subsequent malignancies, age, and year of the diagnosis of a secondary malignancy. SIR describes the relative risk and was defined as the observed count divided by the expected count. It was statistically significant when the *P* value < .05. Confidence limits for SIR were calculated by the methods discussed by Sahai and Khurshid, assuming that the number of observed events followed the Poisson distribution.^14,15^ Excess ratio describes absolute risk and the formula for ER calculation is (observed count − expected count) * 10 000)/person-years at risk.^16^ The expected number, SIR, and excess risk (ER) were calculated using SEER*Stat software. In SEER*Stat, the “unknown” related to treatment depicted the unavailability of evidence in medical records pertinent to chemotherapy and radiation therapy.

### Logistic Regression Analysis and Nomogram

Variables were selected through univariable and multivariable logistic regression analysis to construct the nomogram. We determined a cutoff point of “age at lymphoma diagnosis” by using Receiver Operating Characteristic (ROC) analysis with the Youden index. Time after diagnosis of lymphoma was defined as “intervals between the year of diagnosis and 2018” and divided into groups at 10-year intervals. Univariable logistic regression screened out the related prognostic factors. Then, a multivariable logistic regression analysis was used to analyze the independent risk factors. The variables with a *P* value less than .05 were considered statistically significant.

Based on the factors with a *P* value less than .05 in multivariable logistical regression, a nomogram was established to predict the risk of secondary malignancies for childhood and adolescent primary lymphoma patients. Subsequently, we used a series of methods to validate this nomogram, including the area under the receiver operating characteristic curve (AUC), the calibration curve, and the consistency index (C-index). AUC and C-index were used to assess the discrimination of the model. The calibration curve (1000 bootstrap resamples) was mainly used to test its accuracy. Statistical analyses were performed using SEER (version 8.3.9.2) and R (version 4.0.1).

## Results

### Patient Characteristics

Our study included 5561 patients from the SEER database. Among these patients, 424 developed a secondary malignancy at least one year after the primary diagnosis. The distribution of categorical variables including gender, race, incidence site of lymphoma, histological types, therapy, age at diagnosis, and year of diagnosis was compared between all populations of lymphoma survivors and those who developed a secondary malignancy (Table 1). A higher number of males (57.7%) compared to females (42.3%) among lymphoma survivors was observed, while the number of males (33.0%) was disproportionately less than females (67.0%) among those who developed a secondary malignancy. Among the survivors who developed a second malignancy, classical Hodgkin lymphoma (78.3%) was the most common primary diagnosis. According to the results of the Chi-square test, risk factors that may play a role in the development of a secondary malignancy include sex, age, and year when primary lymphoma was diagnosed, sites, and histological types of primary lymphoma, and therapy strategies.

**Table 1.** Characteristics of patients diagnosed with childhood and adolescent primary lymphoma and those who developed secondary malignancies.

	Primary lymphoma (*N* = 5561)		Secondary malignancies (*N* = 424)		P
	*N*_1_	%	*N*_2_	%	
Sex					<.001
Male	3206	57.7	140	33.0	—
Female	2355	42.3	284	67.0	—
Race					.123
White	4622	83.1	365	86.1	—
Black	599	10.8	43	10.1	—
Other	340	6.1	16	3.8	—
Age at primary lymphoma diagnosis					<.001
01-04	297	5.3	9	2.1	—
05-09	779	14.0	30	7.1	—
10-14	1427	25.7	103	24.3	—
15-19	3058	55.0	282	66.5	—
Year of lymphoma diagnosis					<0.001
1975-1984	1258	22.6	231	54.5	—
1985-1994	1294	23.3	121	28.5	—
1995-2004	1475	26.5	57	13.4	—
2005-2013	1534	27.6	15	3.5	—
Primary lymphoma site					<.001
Nodal	4811	86.5	400	94.3	—
Extranodal	750	13.5	24	5.7	—
Primary lymphoma type					<.001
Classical Hodgkin lymphoma	3233	58.1	332	78.3	—
NLPHL	117	2.1	9	2.1	—
Non-Hodgkin lymphoma, B-cell	1434	25.8	54	12.7	—
Non-Hodgkin lymphoma, T-cell	409	7.4	8	1.9	—
Lymphoid neoplasm, NOS	368	6.6	21	5.0	—
Therapy for primary lymphoma					<.001
Radiation and chemotherapy	1648	29.6	132	31.1	—
Radiation, no/unknown chemotherapy	815	14.7	149	35.1	—
Chemotherapy, no/unknown radiation	2598	46.7	114	26.9	—
No/unknown radiation, no/unknown chemotherapy	500	9.0	29	6.8	—

### Relative and Absolute Risk Analysis of Secondary Malignancies

**Figure 1.**
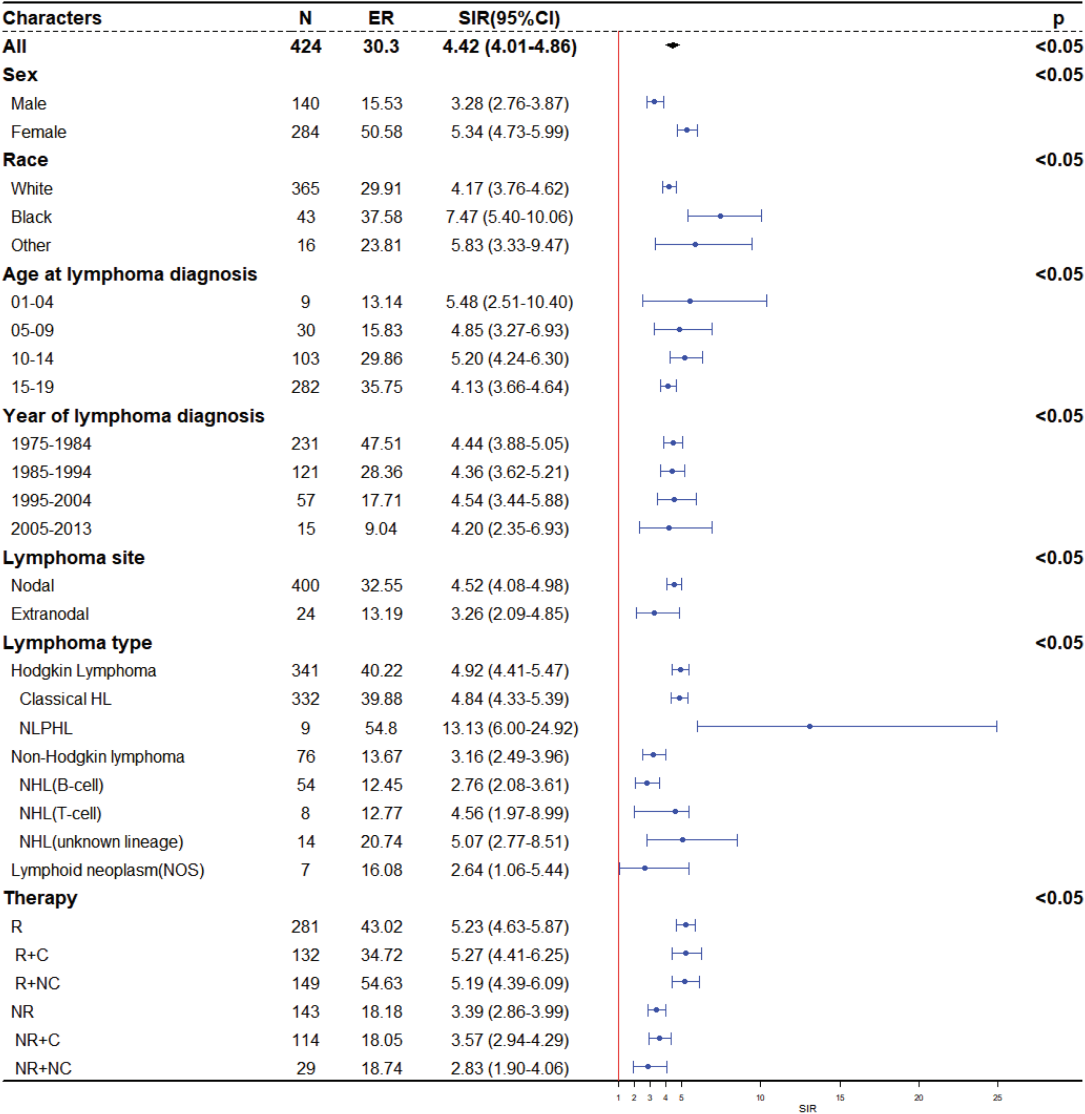
The relative and absolute risk of developing a secondary malignancy for childhood and adolescent lymphoma survivors. Abbreviations: ER: excess risk; SIR: standard incidence ratio; CI: confidence interval; NLPHL: nodular lymphocyte-predominant Hodgkin lymphoma; R: radiotherapy; NR: no/unknown radiotherapy; C: chemotherapy; NC: no/unknown chemotherapy.

In Fig. 1, we showed the relative and absolute risks of developing a secondary malignancy for lymphoma survivors compared to the general US population. The SIR (4.42, 95% CI, 4.01-4.86) and ER (30.29 per 10 000 person-years) describe the relative and absolute risk of developing a secondary malignancy for adolescent lymphoma survivors, respectively, and these values were significantly higher than the general values. Females (SIR = 5.34, 95% CI, 4.73-5.99; ER = 50.58) exhibited a relatively higher SIR and ER than males (SIR = 3.28, 95% CI, 2.76-3.87; ER = 15.53). Blacks were at a higher risk than Caucasians or others. Adolescent Hodgkin lymphoma survivors exhibited a high relative risk and reported >3 times higher absolute risk when compared to NHL survivors. Nodular lymphocyte-predominant Hodgkin lymphoma survivors exhibited typically high SIR (13.13, 95% CI, 6-24.92) and ER (54.79) among all lymphoma types. In addition, lymphoma survivors who underwent radiotherapy, whether they received chemotherapy or not, had typically higher SIR and ER (both radiotherapy and chemotherapy: SIR 5.27, ER 34.72; radiotherapy only: SIR 5.19, ER 54.63) than those who did not receive it.

In addition, we further analyzed the risk of developing a secondary malignancy among specific-type lymphoma survivors who received different treatment strategies (Table 2). Among the patients who received radiotherapy and chemotherapy, extranodal NHL was associated with higher SIR and ER. Survivors who were treated with combined radiotherapy and chemotherapy typically exhibited a higher relative risk when compared to those who adopted other treatment modalities, except for nodal NHL patients. Patients who received radiotherapy and no/unknown chemotherapy had a relatively higher absolute risk when compared to those receiving other treatment modalities, except for extranodal NHL. Nodal HL was associated with the highest risk in cohorts who have not received radiotherapy.

**Table 2.** The relative and absolute risks of developing secondary malignancies among type-specific lymphoma survivors accepted different treatment strategies.

	R+C				R+NC				NR+C				NR+NC			
	*N*	SIR	95% CI	ER	*N*	SIR	95% CI	ER	*N*	SIR	95% CI	ER	*N*	SIR	95% CI	ER
Lymphoma	132	5.27	4.41-6.25	34.72	149	5.19	4.39-6.09	54.63	114	3.57	2.94-4.29	18.05	29	2.83	1.9-4.06	18.74
Hodgkin lymphoma	95	5.51	4.46-6.73	37.37	142	5.25	4.43-6.19	56.28	81	4.57	3.63-5.68	31.01	23	3.17	2.01-4.75	26.54
Nodal	94	5.50	4.45-6.73	37.55	142	5.32	4.48-6.27	57.1	81	4.60	3.66-5.72	31.28	23	3.17	2.01-4.75	26.64
Extranodal	1	—	—	—	-	—	—	—	-	—	—	—	-	—	—	—
Non-Hodgkin Lymphoma	37	4.74	3.33-6.53	29.21	7	4.14	1.67-8.54	33.42	33	2.32	1.6-3.26	7.5	6	2.01	0.74-4.38	7.4
Nodal	25	4.00	2.59-5.91	24.01	6	5.50	2.02-11.97	51.88	25	2.38	1.54-3.52	8.18	4	1.89	0.52-4.85	8.06
Extranodal	12	7.66	3.96-13.38	47.78	1	1.67	0.04-9.32	6.26	8	2.15	0.93-4.24	5.84	2	2.3	0.28-8.3	6.5

The excess risk is per 10 000.

Abbreviations: NR, no/unknown radiotherapy; NC, no/unknown chemotherapy; R, radiotherapy; C, chemotherapy; SIR, standard incidence ratio;CI, confidence interval; ER, excess risk.

In Table 3, we have analyzed the risk of secondary malignancies between different types of secondary malignancies based on two categorical variables: gender and lymphoma types. Adolescent lymphoma survivors were associated with the highest relative risk of developing secondary soft tissue neoplasm (SIR: 12.27, 95% CI, 7.59-18.76), followed by secondary bone and joint neoplasms (SIR: 11.07, 95% CI, 5.52-19.81). Soft tissue neoplasms exhibited the highest SIR among Hodgkin lymphoma survivors, while bone and joint neoplasms showed the highest SIR among NHL survivors. However, the highest absolute risk appeared for breast cancer (ER = 10.09) among Hodgkin and NHL survivors.

**Table 3.** The relative and absolute risks of site-specific secondary malignancies based on gender and lymphoma types among childhood and adolescent lymphoma patients.

Types of SMN	Overall				Male				Female			
	*N*	SIR	95% CI	ER	*N*	SIR	95% CI	ER	*N*	SIR	95% CI	ER
Digestive system	48	4.03	2.97-5.34	3.33	28	3.81	2.53-5.5	3.29	20	4.39	2.68-6.78	3.38
Respiratory system	33	7.60	5.23-10.68	2.65	18	7.31	4.33-11.55	2.48	15	8.00	4.47-13.19	2.88
Bones and joints	11	11.07	5.52-19.81	0.92	5	7.53	2.43-17.56	0.69	6	18.23	6.66-39.68	1.24
Soft tissue including heart	21	12.27	7.59-18.76	1.78	11	10.33	5.15-18.48	1.59	10	15.48	7.41-28.47	2.05
Breast	127	7.15	5.96-8.51	10.09	2	26.58	2.98-95.96	0.31	125	7.07	5.89-8.43	23.52
Urinary system	13	2.63	1.4-4.49	0.74	6	1.72	0.63-3.74	0.4	7	4.82	1.93-9.93	1.22
Brain	14	3.97	2.17-6.66	0.97	10	4.49	2.15-8.26	1.24	4	3.07	0.83-7.87	0.59
Endocrine system	72	7.03	5.5-8.85	5.7	21	8.27	5.12-12.64	2.95	51	6.62	4.93-8.7	9.49
Leukemia	20	5.04	3.08-7.78	1.48	11	4.30	2.14-7.69	1.35	9	6.38	2.91-12.12	1.66
Types of SMN	Primary lymphoma				Primary Hodgkin lymphoma				Primary non-Hodgkin lymphoma			
	N	SIR	95%	ER	N	SIR	95%CI	ER	N	SIR	95%CI	ER
Digestive system	48	4.03	2.97-5.34	3.33	36	4.22	2.96-5.84	4.06	12	3.55	1.83-6.2	2.12
Respiratory system	33	7.60	5.23-10.68	2.65	27	8.34	5.49-12.13	3.52	6	5.45	1.99-11.86	1.2
Bones and joints	11	11.07	5.52-19.81	0.92	5	8.52	2.75-19.88	0.65	6	14.76	5.39-32.12	1.37
Soft tissue including heart	21	12.27	7.59-18.76	1.78	21	18.95	11.73-28.97	2.94	—	—	—	—
Breast	127	7.15	5.96-8.51	10.09	111	7.88	6.48-9.49	14.34	16	4.37	2.49-7.09	3.03
Urinary system	13	2.63	1.4-4.49	0.74	9	2.57	1.17-4.88	0.81	4	2.77	0.75-7.1	0.63
Brain	14	3.97	2.17-6.66	0.97	9	3.97	1.81-7.53	1	5	3.97	1.28-9.26	0.92
Endocrine system	72	7.03	5.5-8.85	5.7	56	7.60	5.74-9.87	7.2	16	5.56	3.18-9.03	3.22
Leukemia	20	5.04	3.08-7.78	1.48	15	5.92	3.31-9.76	1.84	5	3.48	1.12-8.13	0.88

Excess risk is per 10 000.

Abbreviations: ER, excess risk; CI, confidence interval; SIR, standard incidence ratio.

**Figure 2.**
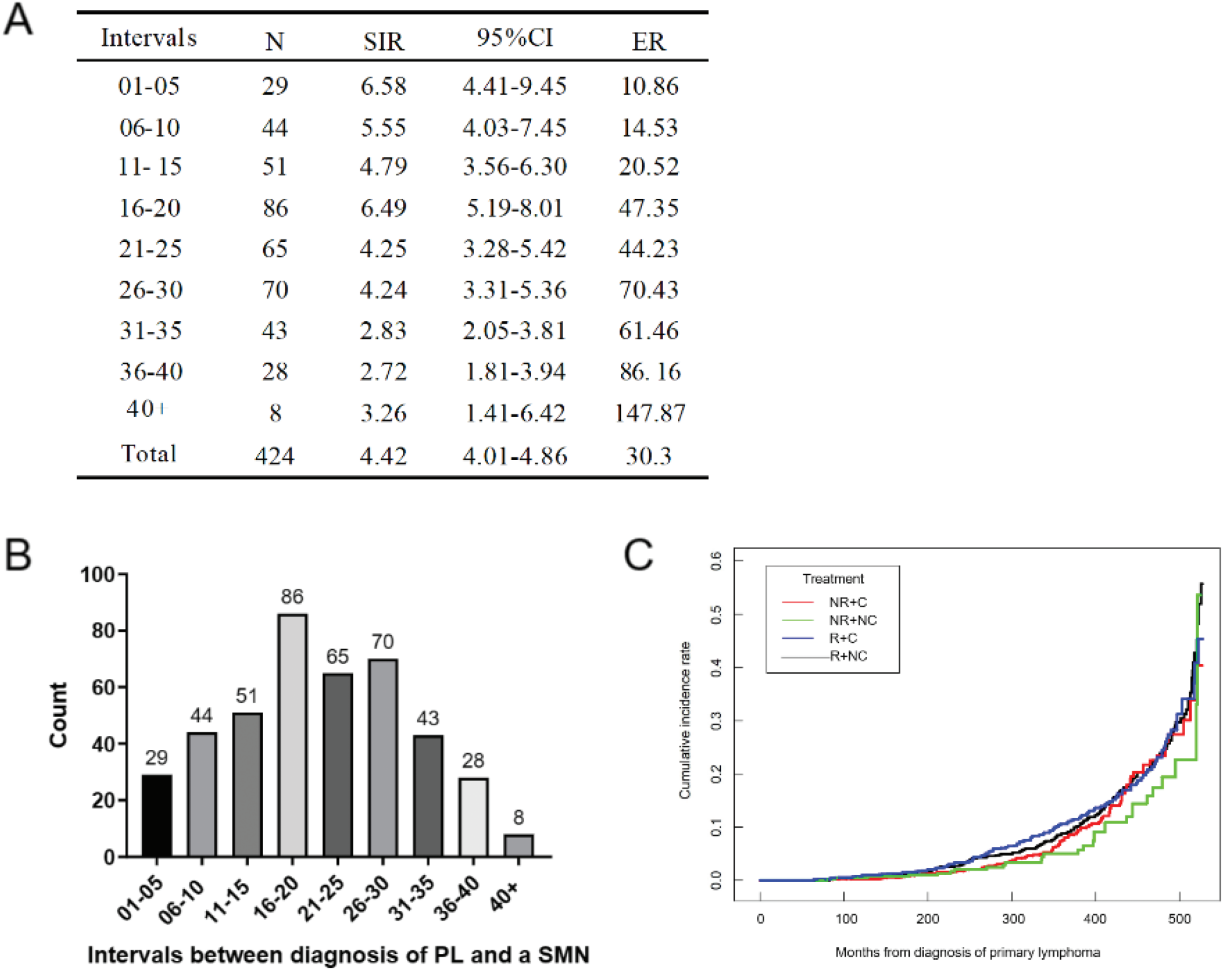
The risk analysis and cumulative incidence rate of a secondary malignancy for lymphoma patients of different intervals between diagnosis of primary lymphoma (PL) and a secondary malignancy (SMN) (*n* = 424). (**A**) The relative and absolute risk analysis of secondary malignancies for lymphoma patients of different intervals between diagnosis of primary lymphoma and a secondary malignancy. (**B**) The distribution of intervals between the diagnosis of primary lymphoma and a secondary malignancy. (**C**) Cumulative incidence rate of a secondary malignancy for primary lymphoma patients with treatment modalities include NR+C, NR+NC, R+C, and R+NC, respectively. Abbreviations: SIR: standard incidence ratio; CI: confidence interval; ER: excess risk; R: radiotherapy; C: chemotherapy; NR: No/unknown radiotherapy; NC: No/unknown chemotherapy.

To elucidate the characteristics of secondary malignancies associated with the duration of lymphoma, the distribution of time intervals between the diagnosis of lymphoma and secondary malignancies and the results of the risk analysis were shown in Fig. 2. The median age of diagnosis for secondary malignancies was 36 years, and the median interval time between the diagnosis of two malignancies was 23 years. Among them, two groups (interval 1-5 years, 16-20 years) were associated with significantly high SIR. ER in various interval groups increased with the length of the interval and ranged from less than 11 to over 147 per 10 000 person-years at risk. Cumulative incidence rate vs. months from initial diagnosis was analyzed by different treatment methods Supplementary Figure 1 but no statistically significant difference was observed (Fig. 2C). The relative and absolute risks of lymphoma survivors who received various treatment modalities in different lymphoma diagnostic year segments were shown in Supplementary Table 1. Their absolute risk was decreasing as the year of diagnosis increased.

### Univariable and Multivariable Analysis

**Figure 3.**
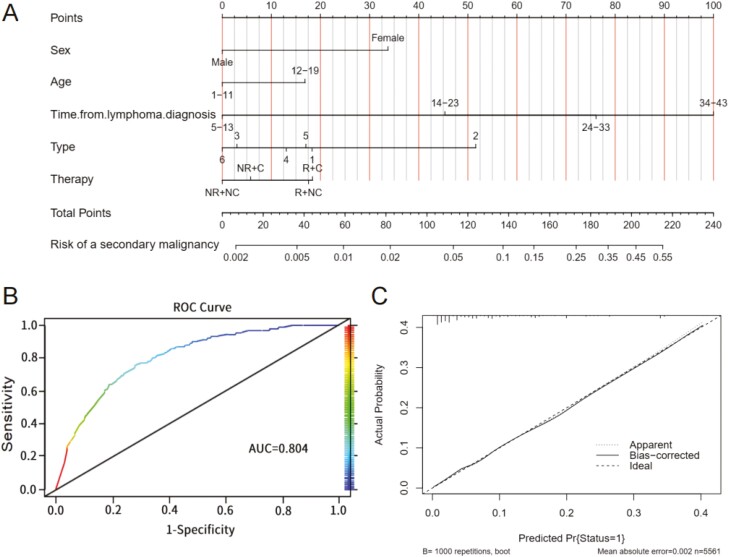
Nomogram constructed for predicting the risk of a lymphoma-related secondary malignancy among childhood and adolescent primary lymphoma patients and its validation. (**A**) Nomogram for predicting the risk of a lymphoma-related secondary malignancy. Each level of different factors corresponds to a point. By adding all these points according to the real condition of a patient, we can obtain a total number that has a corresponding decimal on the “Risk of subsequent cancer” line. Therefore, the risk probability for SMN is (the decimal × 100)%. Mark: 1, classical Hodgkin lymphoma; 2, nodular lymphocyte predominance Hodgkin lymphoma (NLPHL); 3, non-Hodgkin lymphoma, B-cell; 4, non-Hodgkin lymphoma, T-cell; 5, non-Hodgkin lymphoma, unknown lineage; 6, lymphoid neoplasm, none of specific. (**B**) Receiver operating characteristic (ROC) curve. (**C**) Calibration curve. Abbreviations: R: Radiotherapy; C: Chemotherapy; NR: No/unknown Radiotherapy; NC: No/unknown Chemotherapy.

The results of univariable and multivariable logistic regression analysis were reported in Table 4. Univariable analysis revealed that 6 out of 7 variables were associated with the development of secondary malignancies among primary lymphoma survivors. These variables were age, time from lymphoma diagnosis, gender, lymphoma site, types, and therapy. Considering the variables identified in the multivariable analysis, we developed a new nomogram for predicting the risk of lymphoma-related secondary malignancies among childhood and adolescent primary lymphoma patients based on factors including sex, age at diagnosis of primary lymphoma, time from lymphoma diagnosis to 2018, type, and therapy (Fig. 3).

**Table 4.** Univariate and multivariate analysis of secondary malignancies-related risk factors for childhood and adolescent lymphoma survivors.

	Univariable analysis			Multivariable analysis		
	OR	CI	*P*	OR	CI	*P*
Sex						
Male vs. Female	0.33	0.27-0.41	.001	0.35	0.28-0.44	<.001
Race						
White vs. Black	1.11	0.8-1.54	.54	NA	NA	NA
Other vs. Black	0.64	0.35-1.15	.14	NA	NA	NA
Age						
12-19 vs. 1-11	2.54	1.91-3.39	.001	1.68	1.22-2.3	.001
Time from primary lymphoma diagnosis						
05-13 vs. 14-23	0.25	0.14-0.44	.001	0.25	0.14-0.44	<.001
24-33 vs. 14-23	2.57	1.86-3.55	.001	2.57	1.84-3.6	<.001
34-43 vs. 14-23	5.6	4.14-7.56	.001	5.36	3.85-7.45	<.001
Lymphoma site						
Nodal vs. extranodal	2.74	1.8-4.17	.001	1.05	0.65-1.71	.8299
Lymphoma type						
NLPHL vs. CHL	0.73	0.37-1.45	.37	2.78	1.29-5.98	.0088
NHL, T-cell vs. CHL	0.17	0.09-0.35	.001	0.87	0.4-1.9	.7281
NHL, B-cell vs. CHL	0.34	0.25-0.46	.001	0.63	0.45-0.9	.0099
NHL(NOS) vs. CHL	0.51	0.29-0.88	.02	0.97	0.53-1.75	.9165
LN(NOS) vs. CHL	0.58	0.27-1.26	.17	0.58	0.26-1.3	.1866
Therapy						
R+C vs. NR+C	1.9	1.46-2.46	.001	1.47	1.12-1.94	.0054
R+NC vs. NR+C	4.87	3.76-6.31	.001	1.44	1.07-1.94	.0169
NR+NC vs. NR+C	1.34	0.88-2.04	.17	0.84	0.54-1.31	.4368

Abbreviations: NLPHL: nodular lymphocyte predominance Hodgkin lymphoma; CHL: classical Hodgkin lymphoma; NHL, T-cell: non-Hodgkin lymphoma, T-cell; NHL, B-cell: non-Hodgkin lymphoma, B-cell; NHL(NOS): non-Hodgkin lymphoma(NOS); LN(NOS): lymphoid neoplasm (NOS); PL: primary lymphoma; R+C: radiotherapy + chemotherapy; NR+NC: no/unknown radiotherapy + no/unknown chemotherapy.

### Construction and Validation of Nomogram

Each variable was distributed on the nomogram according to its contribution to risk (Fig. 3). The level of the different factors should be identified and then the corresponding number of designated points could be added to the total. For example, being female gender adds 30 points to the total while being male adds 0. By adding all these points to reach a total depending on the real condition of a patient, we can obtain a total point number that has a corresponding decimal on the “Risk of subsequent cancer” line. The risk probability for the incidence of secondary malignancies is (the decimal × 100)%. The nomogram was internally validated using the ROC curve and calibration curve. Nomogram exhibited superior predictive power as indicated by the AUC value of 0.804. Subsequently, we employed 1000 bootstrap resamples to evaluate the accuracy of the nomogram. The concordance index (C-index) was observed as 0.804 and the bias-corrected ­c-index was 0.800. In summary, the predictive plot matched with actual probabilities. The established and validated nomogram is capable of predicting the risk of secondary malignancies among primary lymphoma survivors.

An online version of our nomogram can be accessed at https://zzuxz.shinyapps.io/Nomogram-For-Secondary-Malignancies/ to assist researchers and clinicians in obtaining accurate predictions intuitively and easily by giving clinical features as the significant inputs and subsequently reading output figures and tables generated by the web server.

## Discussion

A previous study reported the cumulative incidence risk of subsequent neoplasms was 26.3% at 30 years after Hodgkin lymphoma diagnosis among 1380 childhood patients diagnosed between 1955 and 1986.^17^ A few reports also described the risk of secondary malignancies such as lung cancer and thyroid cancer among childhood Hodgkin lymphoma survivors.^18^ But, most of these studies only focused on patients of one specific lymphoma type and all age groups, and there were no studies that precisely predicted the risk of secondary malignancies in childhood lymphoma survivors. We selected lymphoma patients diagnosed below 20 years of age who had survived for at least 5 years because the proportion of such survivors has been increasing, and there is a need to unravel their health status after treatment. The current study is a large and comprehensive study that compares the relative and absolute risk of secondary malignancies for childhood and adolescent lymphoma patients in different clinical conditions and identifies risk factors to build a clinically applied predictive nomogram.

In our study, a few of the results pertinent to the incidence of secondary malignancies in primary lymphoma survivors are consistent with previous reports. Gender has been shown to play a crucial role, and female gender is one of the significant risk factors. Older age at the time of diagnosis also enhances the risk of developing secondary malignancies. Hodgkin lymphoma survivors were reported to experience the highest risk of acquiring secondary malignancies. In addition, radiotherapy has elevated the risk of subsequent malignant neoplasms up to 2.7 times (95% CI, 1.0-7.7) among lymphoma survivors of all ages; chemotherapy administration for any primary malignancy also enhances the risk of developing secondary malignancy (SIR = 6.3, 95% CI, 4.1-9.4).^19^

There are several established nomogram prognostic index models depending on the pretreatment indicators to predict the survival conditions of lymphoma patients, which have proven quite useful. Hongyan Li et al described the efficacy of a nomogram to provide a numerical probability of overall survival for patients suffering from extranodal natural killer/T-cell lymphoma.^20^ Jun Cai et al constructed a nomogram to predict the overall survival for patients with diffuse large B-cell lymphoma (DLBCL), which produced a good clinical prognostic stratification for DLBCL.^21^ Analysis of the underlying factors for the risk of secondary malignancies helps clinicians to identify potential risk factors and subsequently enables medical practitioners to design a more ­patient-specific follow-up strategy. This nomogram could be a useful and novel tool to predict the long-term risk of subsequent malignancies for childhood and adolescent lymphoma survivors. The predictors included in this nomogram are considered as significant and easily available in clinical practice.

In this study, we performed a statistical analysis of subsequent malignant neoplasm incidence based on >5000 cases of lymphoma survivors observed throughout 39 years. This study is the first report that explored underlying risk factors associated with the incidence of secondary malignancies in a childhood and adolescent lymphoma patient cohort. We also developed a nomogram to quantitate the risk of secondary malignancies using a simple tool, which was associated with great predictive power. For clinicians, we illustrated the peak time of the onset of secondary malignancies and comparably common types of secondary cancers incidence among lymphoma survivors of different genders and lymphoma types. This nomogram can provide a quantified risk in real clinical practice, for survivors who exhibited a high-evaluated risk, regular, and detailed body examination should be recommended. For patients, knowing the risk of developing another neoplasm may increase their attention to their body and consequently avoid original lifestyle risk factors. However, obtaining this information may also increase the patients’ psychological burden, so we recommend clinicians use this nomogram initiatively but reach a consensus with the patients before telling them the risk of another fatal condition.

However, our study still has some limitations. First, our findings can be applicable to the US population with distinct demographic features, as we procured data from the SEER registries, but these findings need to be further validated in other racially and socially diverse populations. In addition, the nomogram has fewer variables due to the limitation of data availability. The results of laboratory tests and detailed treatment information pertinent to lymphoma should be incorporated into the analysis to further rule out confounding caused by these factors, such as radiation methods or specific types of chemotherapy drugs, older radiation methods are more likely to increase second malignancy risk than new ones. Furthermore, more studies should be conducted to stratify the patients with different risk estimates and develop a matching follow-up and screening strategy to achieve good clinical outcomes. Concomitantly, it is crucial to develop therapies with reliable curative capacity for treating primary lymphomas without carcinogenic potential.

## Conclusion

Lymphoma patients diagnosed before the age of 20 years from 1975 to 2013 and who survived for at least 5 years in the SEER database were selected. We first compared the relative and absolute risk of a secondary malignancy with the general US population and then identified independent risk factors by univariable and multivariable logistic regression analyses, resulting in a quantitative nomogram for predicting the risk of secondary malignancies. This nomogram can help clinicians effectively to identify high-risk patients and raise awareness of recurrent follow-up and screening them in advance for choosing personalized medicine.

Note: Person-time at risk contributed by this subset of your cohort, expressed in years. Person-time at risk: For a particular subject, person-time at risk is the time in years between entry into an analysis and exit from an analysis. This is the time during which a subject is at risk of having an event (whether second cancer, death, etc.) as defined by the analysis. Total person-time at risk for a given subcategory is calculated by adding the person-time at risk counted in that subcategory for each subject in the analysis.

## Supplementary Material

oyad112_suppl_Supplementary_Figure_1

oyad112_suppl_Supplementary_Table_1

## Data Availability

The data underlying this article will be shared on reasonable request to the corresponding author.
