# Supplementary figures and images for: Long-Term Risk of Subsequent Malignant Neoplasms Among Childhood and Adolescent Lymphoma Survivors (1975-2013): A Population-Based Predictive Nomogram
Source: Oncologist. 2023 May 13;28(9):e765–73. doi: 10.1093/oncolo/oyad112 (PMC10485277; doi:10.1093/oncolo/oyad112)

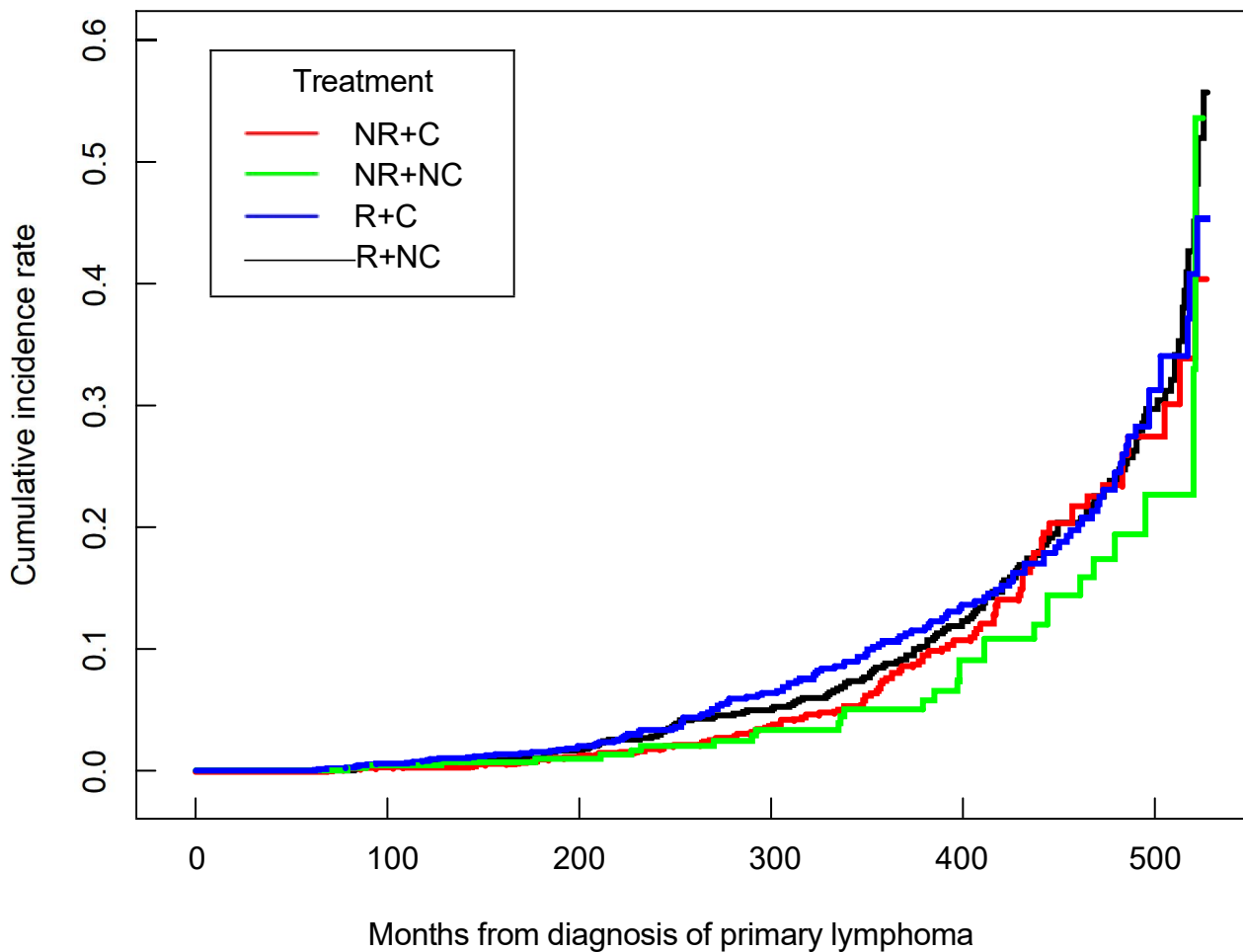

Supplement: oyad112_suppl_Supplementary_Figure_1 [file oyad112_suppl_supplementary_figure_1.pdf]
