# Supplementary material for: Long-Term Risk of Subsequent Malignant Neoplasms Among Childhood and Adolescent Lymphoma Survivors (1975-2013): A Population-Based Predictive Nomogram
Source: Oncologist. 2023 May 13;28(9):e765–73. doi: 10.1093/oncolo/oyad112 (PMC10485277; doi:10.1093/oncolo/oyad112)
Supplement: oyad112_suppl_Supplementary_Table_1 [file oyad112_suppl_supplementary_table_1.docx]

**S-Table1**：

Relative and absolute risk of lymphoma survivors received different treatment in different lymphoma diagnostic year segments.

|  | 1975-1984 | | | | 1985-1994 | | | | 1995-2004 | | | | 2005-2013 | | | |
| --- | --- | --- | --- | --- | --- | --- | --- | --- | --- | --- | --- | --- | --- | --- | --- | --- |
|  | N | SIR | 95%CI | ER | N | SIR | 95%CI | ER | N SIR 95%CI ER | | | | N SIR 95%CI ER | | | |
| NR+NC | 15 | 2.50 | 1.4-4. 13 | 23.77 | 10 | 3.58 | 1.72-6.58 | 24.57 | 4 | 3.49 | 0.95-8.93 | 13.38 | 0 | - | - | -2.74 |
| NR+C | 41 | 3.25 | 2.33-4.41 | 30. 16 | 46 | 4.07 | 2.98-5.43 | 22.95 | 21 | 3.43 | 2. 12-5.25 | 10.8 | 6 | 3. 18 | 1. 17-6.93 | 5.75 |
| R+NC | 116 | 5.40 | 4.45-6.5 | 65.39 | 31 | 4.52 | 3.07-6.42 | 37. 18 | 5 | 5.62 | 1.82- 13. 1 | 32. 18 | 0 | - | - | -3.79 |
| R+C | 62 | 4.94 | 3.79-6.33 | 47.66 | 34 | 5.00 | 3.46-6.98 | 32.61 | 27 | 6. 14 | 4.04-8.93 | 28.6 | 9 | 6.90 | 3. 15- 13. 1 | 18.4 |

Excess risk is per 10,000;

Abbreviations: NR: No/unknown radiotherapy; NC: No/unknown chemotherapy; R: radiotherapy; C: chemotherapy; SIR: standard incidence ratio; CI: confidence interval; ER: excess risk.
